# Na^+^ Binding and Transport: Insights from Light-Driven Na^+^-Pumping Rhodopsin

**DOI:** 10.3390/molecules28207135

**Published:** 2023-10-17

**Authors:** Qifan Yang, Deliang Chen

**Affiliations:** 1 College of Life Sciences, University of Chinese Academy of Sciences, Beijing 100049, China; 2 National Laboratory of Biomacromolecules, Institute of Biophysics, Chinese Academy of Sciences, Beijing 100101, China

**Keywords:** Na^+^ transport, light-driven Na^+^ pumps, microbial rhodopsins

## Abstract

Na^+^ plays a vital role in numerous physiological processes across humans and animals, necessitating a comprehensive understanding of Na^+^ transmembrane transport. Among the various Na^+^ pumps and channels, light-driven Na^+^-pumping rhodopsin (NaR) has emerged as a noteworthy model in this field. This review offers a concise overview of the structural and functional studies conducted on NaR, encompassing ground/intermediate-state structures and photocycle kinetics. The primary focus lies in addressing key inquiries: (1) unraveling the translocation pathway of Na^+^; (2) examining the role of structural changes within the photocycle, particularly in the O state, in facilitating Na^+^ transport; and (3) investigating the timing of Na^+^ uptake/release. By delving into these unresolved issues and existing debates, this review aims to shed light on the future direction of Na^+^ pump research.

## 1. Introduction

Precisely controlled transmembrane transport of Na^+^, an essential element, is vital for sustaining life. Na^+^ mediates crucial physiological processes, including nerve impulse generation and maintenance of electrolyte and fluid balance [1,2,3]. Dysregulation of Na^+^ levels is often associated with various diseases such as heart or kidney failure and stomach cancer [4,5,6,7]. Na^+^ transport proteins can be broadly classified into two categories: Na^+^ pumps and Na^+^ channels [8,9]. Among the pumps, Na^+^ is predominantly transported using energy derived from ATP.

In 2013, the discovery of a light-powered Na^+^-pumping rhodopsin (NaR), *Krokinobacter eikastus* rhodopsin 2 (KR2), unveiled a new member of the microbial rhodopsin superfamily [10]. This novel Na^+^ pump typically comprises seven transmembrane helices that bind a chromophore of a retinal [11,12]. NaR is characterized by a distinct NDQ motif (Asn112-Asp116-Gln123) in its third helix, homologous to the DTD motif (Asp85-Thr89-Asp96) in Bacteriorhodopsin (an H^+^ pump) and the TSA motif (Thr126-Ser130-Ala137) in Halorhodopsin (a Cl^−^ pump) [13,14]. Under physiological conditions, NaR actively transports Na^+^ ions outwards, while it pumps H^+^ in the absence of Na^+^ [10,15,16].

NaR has garnered considerable attention, not only for its potential applications in optogenetics but also as a novel Na^+^ pump [17,18,19]. Extensive data obtained through crystallography, spectroscopic techniques (infrared, Raman, nuclear magnetic resonance), electrical characterization, and other methodologies have provided valuable insights into the light-driven Na^+^ transmembrane transport mechanism. However, notable controversies and inconsistencies persist. This review aims to summarize the current findings, compare the prevailing Na^+^ pumping mechanism with alternative hypotheses, and propose key questions that warrant future exploration. By conducting a comprehensive analysis of three crucial aspects—(1) the pathway for Na^+^ translocation, (2) structural changes, especially in the O state, and (3) the timing of Na^+^ uptake and release—we hope to shed light on the intricate workings of Na^+^ transport mediated by NaR and inspire further investigations in this field.

## 2. Ground-State Structure of KR2

**Figure 1:**
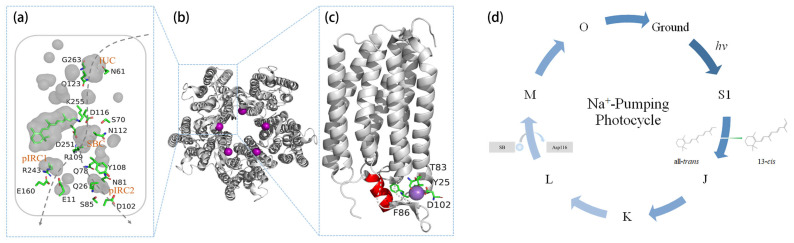
Crystallographic model of KR2 (PDB code: 6REW) in the ground state. (**a**) Illustration of key residues and cavities within the presumed ion transport pathway. (**b**) Top view of the pentamer structure. (**c**) Enlarged side view of a single protomer. (**d**) A typical Na^+^-pumping photocycle model of KR2. Transmembrane helices and cavities are depicted in light gray. Na^+^ and the N-terminal helix are shown in purple and red, respectively. The dotted arrow lines indicate the proposed paths of Na^+^. Relocated Schiff base H^+^ is shown as a blue sphere.

Currently, KR2 serves as a model protein in the study of the NaR family, with the majority of crystal structures reported being of KR2 [11,12,20,21,22]. Under physiological conditions, KR2 oligomerizes as a pentamer on the cell membrane [11,20,21,23]. It incorporates hepta-helical elements into the lipid bilayer and establishes a Schiff base linkage between a conserved lysine residue (Lys255) and a chromophore of an all-*trans* retinal [11,12]. The putative ion translocation pathway primarily comprises three distinct cavities: the cytoplasmic ion uptake cavity (IUC), the central Schiff base cavity (SBC), and the extracellular ion release cavity (IRC) (Figure 1a). In certain pentamer models, an additional Na^+^ ion has been observed bound to the oligomerization interface near the extracellular surface (Figure 1b,c). Additionally, a unique N-terminal helix has been identified (Figure 1c).

### 2.1. Na^+^ Translocation Pathway

The crystal structures of KR2, as reported by several different laboratories, consistently reveal the presence of three distinct regions, forming the putative Na^+^ translocation pathway (Figure 1a). Unlike H^+^ or Cl^−^ pumps, KR2 has an unexpectedly large ion uptake cavity (IUC), which includes residues of Gly263, Asn61, and Gln123. It has been hypothesized that monovalent cations are recruited into IUC from the extracellular side and pass through a filter composed of Asn61 and Gly263, where ion selection based on cation size occurs [12]. In addition, Gln123 also contributes to Na^+^/H^+^ selectivity [14,24]. Studies have shown that mutants with large residues can pump K^+^ (N61P/G263W) and Cs^+^ (N61L/G263F), suggesting that modifying the local structure and expanding the filter size enables the permeation of larger cations [11,12,25]. However, the crystal structure of the G263F mutant contradicts this conclusion, as it exhibits a significantly reduced IUC size, inconsistent with increased K^+^ permeability [20]. This indicates that the ion selectivity of the filter may not be solely dependent on cation size. Most engineered K^+^ pumps have been achieved by the substitution of Gly263 or Asn61 with Tryptophan, Phenylalanine, or Leucine at the entrance to IUC. Thus, a possible interpretation is that these large hydrophobic residues might facilitate the K^+^ dehydration during the uptake [26]. Nevertheless, by employing Kato et al.’s methodology of competitive kinetic analysis, characterizing the K^+^ uptake kinetics in these mutants may provide further detailed explanations for the ion selectivity mechanism of IUC [27,28].

After traversing the central gate formed by the retinal Schiff base and its counterion Asp116, Na^+^ binds to the SBC [21,22]. The functional and spectral changes observed in numerous variants of Asp116, Ser70, Asn112, and Asp251 around this cavity, highlight the crucial role of SBC in ion selectivity and pump activity [10,12,14,21,29,30]. SBC is separated from the extracellular ion release cavity (IRC) by Arg109, which may participate in a hydrogen bonding network and function as an exit gate [31]. Furthermore, R109Q showed passive preferential leakage for K^+^ [32], and S254A near the Schiff base exhibited K^+^ pump activity [20]. This evidence indicates that in the future, improving the ion selectivity for K^+^ or creating new optogenetic tools capable of transporting other cations can be achieved by modifying SBC, in addition to traditionally modifying IUC. 

The pathway of Na^+^ release is currently a subject of controversy. The ground-state structures revealed two putative ion release cavities in the extracellular side of KR2 [11,12,20,21,22]. One is an intra-molecular cavity (pIRC1), primarily surrounded by Glu11, Glu160, and Arg243. The other is an inter-molecular cavity (pIRC2), situated at the pentameric interface and is mainly constituted by Gln78, Asn81, Ser85, Asp102, Tyr108, and Gln26’ (adjacent protomer). While a bound Na^+^ has been resolved on the outer surface of the pentameric interface in the ground state, it is not transferring ions but rather serving to stabilize the pentameric structure [12]. Therefore, the ground-state crystal structures alone could not provide a definitive pathway for ion release. 

Time-resolved serial femtosecond crystallography (TR-SFX) analysis revealed the presence of a positive peak, hypothesized to be a Na^+^, located between Glu11, Asn106, and Glu160, accompanied by the movement of Arg243 at 20 ms after light excitation [22]. Therefore, a prevailing hypothesis suggests that Na^+^ release may occur from the Glu11–Glu160–Arg243 cluster (pIRC1), similar to the H^+^ release from its release complex (Glu194, Glu204, and hydrogen-bonded waters) in bacteriorhodopsin [33,34]. However, mutating Glu11, Glu160, or Arg243 to alanine or polar uncharged residues did not eliminate Na^+^ pumping activity as expected [11,12]. Indeed, it is worth noting that the metadynamics simulations demonstrated that Na^+^ was released into the bulk solution through pIRC2 after passing Arg109 in the majority of simulations (8 out of 10) [21]. However, in two cases, the ions followed a pathway toward pIRC1 before ultimately being released. Currently, the evidence is still insufficient to conclude which cavity, or if both, likely release Na^+^.

The challenges associated with capturing a specific intermediate during the binding of Na^+^ to its release cavity, as well as the limitations of distinguishing Na^+^ from water using X-ray crystallography, pose obstacles to definitively assigning Na^+^ and identifying the transport pathways. Na^+^ has a similar electron density to water molecules, making it difficult to differentiate them solely based on crystallographic data. Therefore, careful functional studies paired with improved structural tools may help resolve these key questions. Due to the advantages of charge detection, which enables the differentiation of Na^+^ from water based on the distribution of electrostatic potential (ESP) [35], cryo-electron microscopy (cryo-EM), particularly time-resolved cryo-EM [36], holds the potential to illuminate the ion release pathway.

### 2.2. Pentamerization

Microbial rhodopsins often assemble into oligomers, but their functional roles exhibit variations [23,37]. Bacteriorhodopsin (BR) serves as an example, forming stable trimers under physiological conditions. Interestingly, BR monomers retain their proton pumping ability, albeit with reduced stability [38]. In contrast, *Gloeobacter* Rhodopsin (GR) experiences a significant distortion of the photocycle during the transition from a trimeric state to a monomer [39]. As for proteorhodopsin (PR), its monomers exhibit approximately five-fold faster photocycle kinetics compared to hexamers [40]. 

As shown in Figure 1b, NaR assembles into a pentamer under physiological lipid environments [11,20,21,23]. Kovalev et al. proposed that the pentameric architecture is essential for the formation of the expanded conformation that allows for Na^+^ passage [20,41]. Additionally, the hypothesized Na^+^ release through pIRC2 suggests that pentameric oligomerization is essential for Na^+^ transport [21]. On the other hand, monomeric KR2 in its crystal form exhibited a typical Na^+^-pumping photocycle kinetics, similar to that of pentameric KR2, indicating that monomers are the functional units for Na^+^ transport. Nevertheless, kinetic similarity alone is insufficient to draw conclusions about Na^+^ transport. Confusingly, the 1.4Å Schiff base opening in the photocycle appears too narrow for Na^+^ (1.9 Å) passage [22], which raises doubts about inferring Na^+^ transport of KR2 in the monomeric form. 

Studies involving mutants, such as H30K and Y154F, which disassemble pentamers into monomers, have suggested that pentamerization plays a critical role in the Na^+^-pumping activity of KR2 [20]. However, the conventional strategy of inducing monomer formation through mutations is not ideal as it poses challenges in distinguishing whether the observed changes in activity are solely attributed to monomer formation or if they are influenced by the direct impact of the mutated residues on the ion transport. Obtaining a stable monomeric form of wild-type NaR in a lipid environment, albeit challenging, would provide a more definitive evaluation of the role of pentamers versus monomers.

### 2.3. Initial Binding of Na^+^

Understanding the mechanism of Na^+^ transport primarily hinges on comprehending the binding of Na^+^. Inoue et al. initially reported the binding of Na^+^ near Asp102 in the B-C loop of KR2 using Fourier transform infrared (FTIR) spectroscopy [10]. This Na^+^, although not transferred during the photocycle, was identified to be coordinated by Tyr25, Thr83, Phe86, and Asp102 from adjacent protomers, and a water molecule at the pentameric interface through crystallography [11]. Recently, a second Na^+^ binding site in the ground state was proposed, which participates in ion transport [21,42].

The binding of Na^+^ exerts notable and intricate effects on the ground-state structure of NaR. Electron paramagnetic resonance (EPR) spectra demonstrated that helices F and G undergo repositioning upon the replacement of K^+^ with Na^+^ [43]. Size exclusion chromatography (SEC) studies indicated that Na^+^ stabilizes pentameric structures [44]. Interestingly, the visible spectrum of KR2 in DDM detergent micelles was reported to be unaffected by Na^+^ [10,43], likely because the binding site is distant from the retinal chromophore in the ground state. However, a ~10 nm redshift in λmax was observed in DDM with Na^+^ compared to K^+^ [44]. Similarly, replacing K^+^ with Na^+^ caused an 8 nm redshift in λmax for DM-solubilized KR2 [43]. Moreover, Raman spectroscopy suggested that Na^+^ may weaken the hydrogen bond between the Schiff base and Asp116 [44], while others found minimal changes among Na^+^, K^+^, and choline^+^ conditions [42]. These contrasting observations need further investigation.

The initial binding of Na^+^ also influences the kinetics of the photocycle. Transient stimulated Raman spectroscopy (TSRS) indicated that extracellular Na^+^ binding significantly affects the equilibrium between the K/L/M intermediates [45]. Low-temperature Raman spectroscopy of *Ia*NaR revealed a notable dependence on cations in the K state, with a decrease in chromophore distortion near the Schiff base associated with lower cation binding affinity [42]. Previous light-induced FTIR difference spectroscopy of KR2 at 77 K did not detect such structural differences in the K state between NaCl and KCl [46], potentially due to obscuration by protein spectral changes. 

Initial binding of Na^+^ may greatly impact NaR structures in both the ground state and photocycle. Unfortunately, the absence of crystals grown in Na^+^-free precipitants at physiological pH hampers a direct comparison between structures with and without initial Na^+^ binding. Moreover, several unanswered questions persist, including whether initial Na^+^ binding is a prerequisite for Na^+^ transport and whether Na^+^ binding cooperatively occurs among protomers in the pentameric structure.

### 2.4. N-Terminal Helix

The role of the unique N-terminal helix has been largely overlooked in the NaR field. According to Kato et al., the N-terminal helix of KR2 is primarily involved in maintaining the structural stability of the protein rather than contributing to its pumping activities [12]. Interestingly, when the N-terminal was modified in KR2 (eKR2), it acquired the ability to transport K^+^ [47]. Cl^−^ pump rhodopsin (ClR) also possesses an N-terminal helix, but studies on its role are scarce [48]. Furthermore, a rhodopsin phosphodiesterase (Rh-PDE) was found to have an additional N-terminal transmembrane domain (TM0) that is crucial for its enzymatic activity [49]. The N-terminal helix is not a common feature among known microbial rhodopsins. However, as more diverse members of the rhodopsin family are discovered in the future, investigating the structural and functional roles of the N-terminal helix from an evolutionary perspective can offer valuable insights.

## 3. Na^+^-Pumping Photocycle

The illumination induces an initial *trans*-to-*cis* photoisomerization around the C13=C14 bond in the retinal chromophore, initiating the photocycle (Figure 1d). During the recovery of the retinal to its initial configuration, a limited number of quasi-stable intermediates (K, L, M, and O) transiently populate [10,19,50]. Notably, Inoue et al. proposed a photocycle model in 2013 [10], which has since been expanded and refined by subsequent studies. Hontani et al. introduced the J, K/L_1_, and K/L_2_ states into the photocycle [45], while Kajimoto et al. identified the intermediates O_1_ and O_2_ [51]. Eberhardt et al. proposed a temperature-dependent equilibrium composition of the K, L, and M intermediates, with an irreversible M to O transition [52]. Skopintsev et al. added a K_L_ state before the L/M equilibrium [22]. Recently, Kato et al. revealed the presence of seven intermediates and proposed an accessibility switch between the O_1_ to O_2_ transition [53].

### 3.1. Initial Steps of Retinal Isomerization

The initial event in the photocycle is the absorption of a photon, which gives rise to reactive and non-reactive excited states of the chromophore. KR2 exhibits the generation of multiple S1 states due to ground-state structural inhomogeneity, as observed in studies by Tahara et al. [54,55]. This observation may be attributed to the presence of two conformations of the Schiff base counterion Asp116 at physiological pH, as revealed by the crystal structure (PDB code: 3X3C) [12]. Molecular dynamics simulations and large-scale XMCQDPT2-based QM/MM modeling further support these findings, linking KR2’s reactive and non-reactive states to three distinct retinal pocket conformations [56]. While this specific topic lies beyond the scope of our current discussion, a comprehensive review by Asido et al. provides further insights [57]. 

For the functionality of KR2 and most microbial rhodopsins, photon absorption triggers retinal isomerization, resulting in the formation of a J state (Figure 1d), the first photoproduct, from the S1 excited state and a fully 13-*cis* retinal [54]. The transition from S1 to the J state occurs with a time constant of 180 fs, which is three times faster than the corresponding process in BR [58,59]. This difference is attributed to the presence of a strong and direct Schiff base-counterion hydrogen bond in KR2 [56], as opposed to a water-mediated Schiff base-counterion hydrogen bond in BR [60,61]. Subsequently, a blue shift of approximately 30 nm in the photoproduct band, indicates the conversion from the J state to the K state, a process occurring with a time constant of 500 fs [54]. 

### 3.2. Relocation of Schiff Base H^+^

The formation of the K state induces a greater localized distortion of the retinal chromophore near the Schiff base, compared to the ground state [46]. Concurrently, the hydrogen bonding network in the cytoplasmic Gln123/Ser64 region weakens, potentially priming the cytoplasmic channel for the subsequent passage of Na^+^ ions [62].

The subsequent L state reveals more intricate structural changes than those observed in the K state. These changes are believed to facilitate the initial relocation of the Schiff base H^+^. While the retinal chromophore undergoes structural relaxation [45], it also twists more significantly than in the ground state [63]. This out-of-plane twist positions the Schiff base H^+^ in a more favorable manner with respect to Asp116, a prerequisite for the subsequent H^+^ transfer during the L to M transition. However, there is a discrepancy concerning the location of this twist. One viewpoint suggests that it occurs at the mid-polyene chain, deviating from its proximity to the Schiff base in the ground and K states [64]. Conversely, studies using transient absorption/resonance Raman spectroscopy in *Ia*NaR reveal that the retinal chromophore distortion is still near the Schiff base [51]. Solid-state NMR analysis also supports the finding of a twist near the C14–C15 Schiff base region in KR2’s L state [63].

Another crucial structural alteration involves the shortening of the Schiff base-Asp116 distance [22,45,63], leading to increased hydrogen bonding strength and a lowered barrier for proton transfer directly from the Schiff base to its counterion, Asp116. This contrasts with the process observed in BR, where the Schiff base proton transfers to Asp85 via water (wat402) [65]. Additionally, the C20 methyl group of the retinal pushes Val117, ultimately resulting in the flipping of Val117 and inducing structural changes in helix C [22]. These changes are believed to play a role in transmitting the energy from light into the helical bundle, thereby fueling more significant conformational changes at later stages.

In the subsequent M state (Figure 1d), significant changes occur. The Schiff base proton relocates to Asp116, resulting in a further relaxation of the retinal chromophore [45,66]. This relocation is accompanied by the flipping of protonated Asp116 to Ser70/Asn112, forming hydrogen bonds with Asn112 [12,66]. It is noteworthy that while Kato et al. observed this flip in acid-soaked crystals [12], representing an M-like state, Kovalev et al. did not observe the same flip in their acid-soaked crystals [20].

On the cytoplasmic side, the M state features the formation of an open cytoplasmic half-channel, which recruits Na^+^ to the Gly263/Asn61 cytoplasmic IUC [11,12]. However, this commonly accepted notion contrasts with molecular dynamics simulations based on the KR2 crystal structure 3X3B, which indicates that Na^+^ has already reached the cavity of Ser60/Ser64/Gln123 in the K/L state [67]. It is important to note that, to date, the definitive structure of the M state has not been reported through crystallography. Trapping a sufficient population of M-state structures in the crystal, while minimizing the accumulation of O- or L-state structures, presents a challenge during sample illumination. To address this challenge and enable future structural determination, potential approaches include mutant screening and rational design on NaRs. These methods could help identify a candidate with significantly slowed kinetics in the M state, facilitating the trapping of a dominant M-state population for structural analysis. 

### 3.3. Transient Na^+^ Binding near the Schiff Base

**Figure 2:**
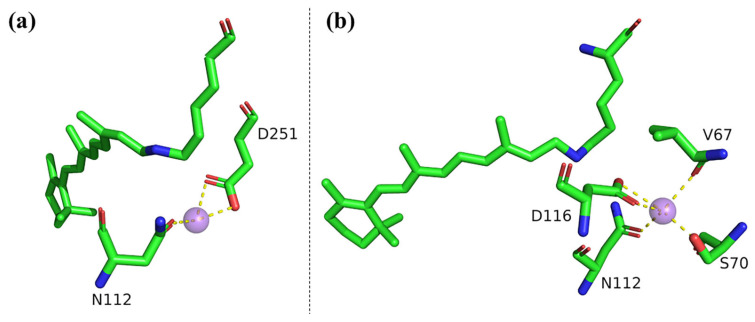
Key structural differences around the Schiff base cavity in the putative O-state crystal structures of 6TK2 and 6XYT. (**a**) In 6TK2, the retinal adopts a 13-*cis* configuration, and Na^+^ is coordinated by Asn112 and Asp251. (**b**) In 6XYT, the retinal is in an all-*trans* configuration, and Na^+^ ions are coordinated by Val67, Ser70, Asn112, and Asp116. Na^+^ is represented as purple spheres, while coordinated water molecules are not depicted.

During the M to O state transition, Na^+^ traverses the intracellular half-channel and binds near the Schiff base region, leading to the formation of the O state (Figure 1d). Understanding the Na^+^-pumping mechanism relies on elucidating the O-state structure [18,68]. Two crystallographic structures, 6TK2 and 6XYT, have been reported for the O state, but controversies exist regarding the Na^+^ binding site and retinal configuration (Figure 2). In 6TK2, the retinal adopts a 13-*cis* configuration, and Na^+^ is coordinated by Asn112 and Asp251 [22]. Conversely, in 6XYT, the retinal is in an all-*trans* configuration, and Na^+^ is coordinated by Val67, Ser70, Asn112, Asp116, and a water molecule [21]. Several aspects of the experimental methods and data statistics are presented below for further comparison.

Regarding the experimental methods, Skopintsev et al. excited the monomeric form of KR2 (6TK2) in the crystals using 575 nm femtosecond laser pulses, achieving a 14% activation level at room temperature to determine the O-state structure [22]. In contrast, Kovalev et al. illuminated the pentameric form of KR2 (6XYT) in the crystals with continuous 532 nm laser light, resulting in the 100% trapping of the O state at 100 K [21]. They also found that the structure trapped at 100 K was identical to that trapped at room temperature, ruling out artifacts during cryo-trapping. For accurate and reliable intermediate structure determination, achieving a higher activation level and dominant occupancy of the intermediate of interest is desirable.

Regarding the data statistics (Table 1), several parameters were compared: (1) Resolution: The reported resolution of 6TK2 is 2.5 Å, while 6XYT has a resolution of 2.1 Å. Higher resolution in protein crystal diffraction reflects finer details, such as bound ions or water molecules, with increased accuracy. (2) R-free: The R-free value for 6TK2 is ~32%, while for 6XYT, it is ~20%. This factor measures the agreement between the experimental raw data and the simulated diffraction pattern calculated from the refined structure model. A lower R-free value indicates better agreement between the model and the experimental data. (3) B-factor: The B-factor represents the extent of atomic displacement around their average positions. Higher B-factors indicate larger atomic vibrations and greater positional uncertainty, while lower B-factors suggest accurate atomic positions and good crystal quality. As shown in Table 1, the overall B-factors of 6TK2 (protein, water, retinal, and Na^+^) are slightly smaller than those of 6XYT. It is worth noting that due to the coordinating atoms being in close proximity to the central Na^+^ and forming stable chemical bonds with it, their thermal vibrations are expected to be similar to those of Na^+^. However, the B-factor values of the coordinating atoms in 6TK2 are significantly smaller than that of Na^+^. In 6XYT, there is less disparity between the B-factors of the coordinating atoms and Na^+^.

Furthermore, the coordination complex of Na^+^ with the surrounding residues follows certain rules, such as the coordination number, geometry, distances, and angles. Both models exhibit octahedral geometry and appropriate distances, but in 6TK2, the ligand-metal-ligand (L-M-L) angles deviate slightly from the ideal geometry. In 6XYT, Na^+^ is coordinated by six atoms, akin to intrinsic hydrated Na^+^, while 6TK2 coordinates Na^+^ with only three atoms. 

Regarding the controversies surrounding the retinal configuration, previous studies using FTIR [69], Raman spectroscopy [64], and SAC-CI calculations [70] supported the 13-*cis* configuration, while MD simulations [67], DNP-NMR [63], and an earlier FTIR study [30] favored the all-*trans* configuration.

To reconcile the discrepancies between the two models, Skopintsev et al. hypothesized that 6XYT represents a later O state compared to the O state represented by 6TK2 because the retinal configuration in 6XYT is all-*trans*, similar to the ground state. In other words, Na^+^ may initially bind to Asn112 and Asp251 and then move to Asp116 in the O state. However, Skopintsev’s 13-*cis* configuration was sustained up to 20 ms, even when the O state substantially decayed, and they did not observe a later O state with an all-*trans* retinal [22]. Conversely, metadynamics simulations by Kovalev et al. suggested that Na^+^ first binds to Asp116 and then moves to Asn112 and Asp251 [21]. Therefore, the two models appear fundamentally incompatible. Further persuasive evidence from other techniques, particularly cryo-electron microscopy [35], which can distinguish positively charged Na^+^ from water molecules, holds the potential to resolve the remaining contentious aspects. 

On the other hand, while the crystal structure of the intermediate provides direct evidence, it is equally crucial to consider findings obtained through alternative methodologies. The theoretically calculated absorption wavelengths for the XRD-O (same as 6XYT) and XEFL-O (same as 6TK2) structures using quantum mechanics/molecular mechanics (QM/MM) approaches are consistent with the experimentally measured ~600 nm, suggesting that both conformations represent the O state [71]. Additionally, Asido et al. measured transient absorption changes in the near-ultraviolet region, revealing an overlap between signals originating from the 13-*cis* and all-*trans* retinal during the rise and decay of the O intermediate in KR2 [72]. This indicates the retinal may re-isomerize from 13-*cis* to the all-*trans* form during the transition process. Furthermore, utilizing time-resolved Raman spectroscopy in *Ia*NaR, Fujisawa et al. observed two O intermediates (O_1_ and O_2_) containing 13-*cis* and all-*trans* retinal, respectively, with an irreversible transition from O_1_ to O_2_ [53,73]. These results strongly suggest that the O intermediate can exist as two distinct structural states, namely the 13-*cis* or all-*trans* forms. The structural information presented provides reasonable evidence to suggest Na^+^ may be sequentially transferred during the transition between the two types of O intermediate.

### 3.4. The Timing of Na^+^ Release

**Figure 3:**
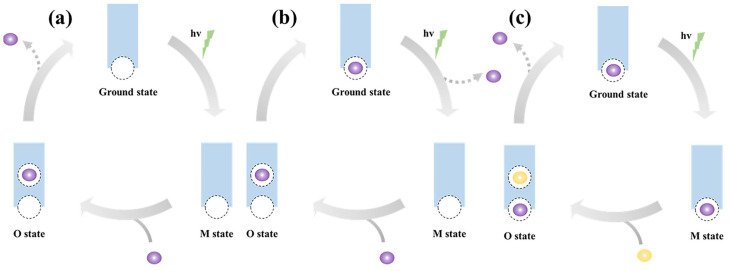
Three tentative schemes for the timing of Na^+^ uptake/release. (**a**) The Na^+^ release site is vacant in the ground state. Na^+^ is taken up during M decay, bound to the upper binding site (Schiff base region) in the O state, and released upon the recovery of the initial state. (**b**) The Na^+^ release site is occupied by Na^+^ in the ground state. While excited, this Na^+^ would be released to the bulk before M decay. Subsequently, another Na^+^ is taken up, moves to the vacant release site, and remains there until the next excitation. (**c**) An initial Na^+^ (purple) bound at the Na^+^ release site in the ground state. When M decays, another Na^+^ (yellow) is taken up and bound at the upper site in the O state. During O decay, the taken Na^+^ moves to the lower site and replaces the initial Na^+^. For the sake of clarity, only the ground, M, and O states in the Na^+^-pumping cycle are shown. Na^+^ is visually represented as a sphere. The dashed circle is used to indicate a postulated Na^+^ binding site. The upper circle denotes a transient site near the Schiff base that forms in the O state, while the lower circle represents a putative Na^+^ extracellular release site.

During the transition from the O state to the initial state, the retinal molecule reverts to its ground configuration, and Na^+^ dissociates from the Schiff base region. In the field of NaR, the prevailing view is that Na^+^ is released to the extracellular solution following its uptake (Figure 3a). This mainstream hypothesis was further systematically summarized and improved by Kandori et al., who proposed a modified Panama Canal model to explain the Na^+^ transport mechanism [18]. In their model, Kandori et al. more comprehensively and in detail described the initial energetically stable state of the unbound substrate Na^+^, the subsequent Na^+^ uptake and binding states caused by the energy input, and the final Na^+^ release state [18]. Therefore, Kandori et al. proposed that NaR is a unique active transporter that does not initially bind the substrate Na^+^ in its resting state [18]. Na^+^ is taken up from the cytoplasmic side during the M to O transition and subsequently released to the extracellular side during the O decay. Thus, their view on the timing of Na^+^ uptake/release is entirely consistent with the mainstream model (Figure 3a). However, due to the lack of crystal structural evidence, the precise timing of Na^+^ release remains uncertain.

In the case of well-studied light-driven H^+^ and Cl^−^ pumps, the kinetics of ion uptake and release are known to depend on the concentrations of the transporting ions [74,75]. For example, in bacteriorhodopsin, the release of protons to the bulk solution from its extracellular release complex is inhibited by higher proton concentrations (pH < 6) [76]. However, the O decay process of NaR appears to be independent of Na^+^ [77], which challenges the hypothesis that Na^+^ is released to the extracellular solution during the O decay. This raises two possibilities: either the Na^+^ release site has an extremely low affinity for Na^+^ during release, making it insensitive to inhibitory effects of high environmental Na^+^ concentrations, or Na^+^ is not released to the bulk solution during the O decay but instead moves outward from the Schiff base region and remains at the ion release site.

The mainstream hypothesis that Na^+^ release follows uptake has additional flaws. It was initially proposed based on the absence of transporting Na^+^ in the initial ground state of KR2. Although a Na^+^ binding site near Asp102 at the pentameric interface was identified through crystallography and FTIR studies, the D102N mutant, which lacks this binding, still retains pump activity. Therefore, it was suggested by Inoue et al. that this Na^+^ binding is not involved in ion transport [12]. However, relying solely on mutation experiments to infer that the interface-bound Na^+^ is non-transportable is not sufficiently convincing. The presence of transporting ions bound to the pumps is not always necessary for maintaining transport activity. For example, in the case of a Cl^−^ pump, the transporting Cl^−^ binds at the cytoplasmic surface, but removing its binding by mutation does not affect pump activity [48]. Furthermore, recent studies have provided evidence for additional Na^+^ binding sites [21,42], suggesting a more complex ion binding and release mechanism. Additionally, the K to L/M transition displays Na^+^-dependent kinetic changes [53], which could potentially suggest that Na^+^ movement occurs even before Na^+^ uptake during the M decay. These observations support the possibility of early Na^+^ release as an explanation.

The presence of Na^+^ bound in the ground state does not definitively determine the sequence of Na^+^ uptake and release. Two alternative models are proposed, both involving initial Na^+^ binding. The first model suggests that Na^+^ release to the extracellular surface occurs before Na^+^ uptake from the cytoplasmic surface (Figure 3b). This mechanism is similar to the established process in bacteriorhodopsin (BR), where H^+^ release to the extracellular surface is linked to the M2 to M2’ transition, followed by reprotonation of Asp-96 from the cytoplasmic surface during the N to N’ transition [78]. Alternatively, an exclusion mechanism has been proposed [21]. According to this model, the Na^+^ taken up during the M decay displaces the initial extracellular Na^+^ and leads to its release during the O decay (Figure 3c).

In the study of the H^+^ pumping mechanism in bacteriorhodopsin, direct measurements of H^+^ kinetics using H^+^-selective dyes, like pyranine, have significantly contributed to understanding the timing of H^+^ release/uptake [78,79]. However, the lack of sensitive and specific Na^+^ dyes has hindered the direct detection of Na^+^ kinetics using Na^+^-selective dyes. An alternative technique, the time-resolved photoelectric response, has been applied to elucidate Na^+^ release/uptake in the NaR family [68,77]. However, extracting the electric signal originating from Na^+^ and removing interference from other ions at the electrode interface poses a significant challenge. Advancements in the development of specific Na^+^-selective dyes and electrodes could greatly enhance the exploration of Na^+^-pumping kinetics and mechanisms.

## 4. Conclusions

Na^+^ transport across the membrane presents several challenges, including the need for input energy, the hydrophobic membrane environment, and the presence of protons producing energy barriers in the ion transport pathway. To overcome these challenges, light-driven Na^+^ pumps have employed several strategies. An all-*trans* retinal is linked as a light-trapping chromophore to power conformational changes enabling Na^+^ transport; hydrophilic residues align to form Na^+^ pathways through the hydrophobic membrane, overcoming the unfavorable hydrophobic environment; Asp116 attracts the Schiff base proton, diverting it from the transport pathway to reduce barriers and enable Na^+^ recruitment. With Na^+^ uptake and passage through the intracellular half-channel, H^+^ reassociates with the Schiff base to prevent the reverse flow of Na^+^. In summary, a comprehensive understanding of its detailed mechanism awaits advancements in detecting Na^+^ transporting kinetics and resolving key structural uncertainties. Further research on NaR is necessary to uncover the intricacies of how it utilizes light energy to induce protein structural changes, facilitating unidirectional Na^+^ transport across membranes. 

As a novel family of Na+ pumps, NaR serves as an illuminating reference for the fundamental processes involved in Na+ transport, providing valuable insights that can inform subsequent mechanistic studies and find applications in a broader range of Na+ pumps or channels.

Many of the previously mentioned structural and functional research findings were achieved through extensive studies of mutations in NaR. To aid interested readers in understanding the relevant studies discussed in this manuscript, we summarized the key findings and conclusions from the vast majority of mutations published in the NaR field and compiled them into Table 2. We also hope that this centralized collection of insights from mutagenesis research will benefit readers through convenient reference.

## Figures and Tables

**Table 1:** Selected crystallography data statistics of the O state. Two structural models, 6TK2 and 6XYT, are compared. For the sake of simplicity of comparison, the 6XYT B-factors listed are averaged from 5 monomers in the pentamer. The water is not listed in the coordinating atoms of Na^+^.

	**6TK2**		**6XYT**	
Resolution (Å)	2.5		2.1	
R-free (%)	32.4		20.0	
	B-factor (Å^2^)			
Protein	41.7		58.9	
Water	43.6		62.2	
Retinal	36.7		56.9	
Na	47.0		57.3	
Coordinating atoms of Na^+^	Asn112-OD1	28.9	Val67-O	48.0
	Asp251-OD1	35.0	Ser70-OG	49.8
	Asp251-OD2	38.2	Asn112-OD1	52.6
			Asp116-OD1	54.5
			Asp116-OD2	62.0

**Table 2:** Major mutations and the corresponding findings. Some mutants without special changes are not listed.

Mutant	Description	Mutant	Description
E11A	Na^+^ pump activity lowered [11,12] Unstable [11,12]	Y25F	Na^+^ binding abolished [80]
H30A	H^+^ pump activity abolished [10] Photocycle slowed [81]	H30L	Na^+^ pump activity abolished [20] Unstable [20]
H30K	Na^+^ pump activity lowered [20] Pentamerization disrupted [20]	L32E	Leaky pump [32]
N61P	Na^+^ pump activity lowered [25]	N61L	K^+^ pump activity [25]
N61M	Similar to N61P [11]	N61Y	K^+^/Cs^+^ pump activity [12,25]
N61W	Pump activity abolished [25]	S64A	Pump activity lowered [62]
S70A	Na^+^ pump activity abolished [12] Photocurrents lowered [32]	S70V	Similar to S70A [32]
S70T	Na^+^ pump activity lowered [12]	L74A	Na^+^ pump activity lowered [21]
L75A	Na^+^ pump activity lowered [21]	L75K	Leaky pump [32] pKa of D251 increased [32]
Q78A	Na^+^ pump activity lowered [21]	Q78L	Function retained [21]
Q78Y	Similar to Q78A [21]	Q78W	Similar to Q78A [21]
D102N	Na^+^ binding abolished [12,80] Thermostability reduced [12] Equilibrium in K/L/M shifted [45]	D101N (*Ia*NaR, D102 in KR2)	Na^+^ binding retained [42]
Y108A	Pentamerization retained [81] Pump activity abolished [21]	R109A	Pump activity abolished [10,31,32] Na^+^ binding abolished [10] K intermediate only [31]
R109K	D116-SB interaction weakened [31] Pump activity lowered [31]	R109N	Leaky pump [32] Pump activity abolished [32]
R109Q	Passive ion conductance [32] Residual Na^+^ pump activity [32] K intermediate prolonged; L/M absent [32]	R109Q (NdR2)	Leaky pump [32]
R108Q (NMR2, R109 in KR2)	Leaky pump [32]	R108Q (*Ia*NaR, R109 in KR2)	R108–D250 interaction broken [42]
N112G	Na^+^ pump activity lowered [29]	N112A	Na^+^ pump activity abolished [10,12,29] O intermediate absent [14,29]
N112S	Similar to N112G [29]	N112C	Na^+^ pump activity abolished [29]
N112P	Na^+^ pump activity abolished [29]	N112D	Na^+^ binding abolished [10] Na^+^/H^+^ pump activity lowered [29]
N112T	K intermediate prolonged; O absent [29] Na^+^ pump activity lowered [29]	N112V	Na^+^ pump activity abolished [29] O intermediate accumulated [29]
N112E	Na^+^ pump activity abolished [29]	N112Q	Similar to N112P [29]
N112H	Pump activity abolished [29] K intermediate only [29]	N112L	Similar to N112P [29]
N112I	Similar to N112P [29]	N112M	Similar to N112P [29]
N112F	Similar to N112P [29]	N112K	Similar to N112H [29]
N112Y	Pump activity abolished [29]	N112R	Similar to N112Y [29]
N112W	Na^+^ pump activity abolished [29] H^+^ pump activity lowered [29]	N112D (NdR2)	Na^+^ binding abolished [82] O intermediate accumulated [82] Photocycle slowed [82]
D116A	Pump activity abolished [10] Red-shifted absorption peak [10]	D116N	Pump activity abolished [10] Red-shifted absorption peak [10,32] Red-shift intermediate only [10,72]
D116E	Na^+^ pump activity abolished [10] Weak Glu-SB hydrogen bond [31]	D116T	Pump activity abolished [83]
D116N (NdR2)	Red-shifted absorption peak [82]	D116T (NdR2)	Red-shifted absorption peak [82] 9-cis-retinal [82]
D116N (GLR)	Red-shifted absorption peak [30] Unstable at pH below 3.5 [30]	D115N (*Ia*NaR, D116 in KR2)	HOOP intensity of K intermediate decreased [42]
D101N (*Be*NaR, D116 in KR2)	Red-shifted absorption peak [84] Pump activity abolished [84]	Q123A	Na^+^ pump activity lowered [10] Na^+^ uptake slowed [14]
Q123V	Na^+^ uptake slowed [14]	Q123D	H^+^ pump activity enhanced [10]
Q123E	Similar to Q123D [10]	Q123D (*Dokdonia* sp. PRO95)	Na^+^ pump activity lowered [24] H^+^ pump activity [24]
Q123E (*Dokdonia* sp. PRO95)	H^+^ pump [24]	Y154A	Pentamerization disrupted [81]
Y154F	Na^+^ pump activity lowered [20] Pentamerization disrupted [20]	E160A	Na^+^ pump activity lowered [11,12] Unstable [11,12]
E160Q	Unstable [10]	P219R	Pump activity lowered [85] Blue-shift absorption peak [85]
R243A	Na^+^ pump activity lowered [11,12] Unstable [11,12]	R243Q	Similar to R243A [11]
D251A	Pump activity abolished [10]	D251N	Similar to D251A [10,32]
D251E	Pump activity abolished [10] Leaky pump [32]	D251N (NdR2)	Photocycle slowed [82] O-like intermediate absent [82]
D251E (GLR)	Na^+^ binds [30] Red-shifted absorption peak [30]	D250N (*Ia*NaR, D251 in KR2)	R108–D250 interaction broken [42]
D230N (*Be*NaR, D251 in KR2)	Pump activity abolished [84]	C253S (*Dokdonia* sp. PRO95)	H^+^ pump activity [86]
S254A	Red-shifted absorption peak [87] K^+^ pump activity [20]	K255A	Na^+^ pump activity abolished [88]
K255G	Na^+^ pump activity abolished [88] O intermediate absent [88]	G263L	Pump activity lowered [11]
G263F	Na^+^ pump activity lowered [11] H^+^ pump activity abolished [11] K^+^/Cs^+^ pump activity [25]	G263W	K^+^ pump activity [12]
N61Y/G263W	Pump activity abolished [12]	N61P/G263F	K^+^ pump activity [25]
N61Y/G263F	Similar to N61P/G263F [25]	N61P/G263W	K^+^ pump activity over Na^+^ [12,25]
N61L/G263F	K^+^/Cs^+^ pump activity [25]	N61L/G263W	Similar to N61Y/G263W [25]
N61P/G263W (NdR2)	K^+^ pump activity [28] Na^+^ affinity decreased [28]	S70A/R109Q	Pump activity abolished [32] Leaky pump [32]
F72G/D116T	Cl^−^ pump [83]	F72G/D102N/ D116T	Cl^−^ pump activity enhanced [83] Cl^−^-dependence color [83] Red-shifted intermediate only [83]
E90Q/E91Q/ D98N/D102N	Na^+^ binding abolished [10]	D102N/N112D/D116T/Q123D	Na^+^ pump activity abolished [83]
D102N/N112D/D116T/Q123E	Similar to D102N/N112D/D116T/Q123D [83]	D102N/D116T	Cl^−^ pump [83] Cl^−^-dependence color [83]
R109Q/D251N	Pump activity restored [32]	R109Q/D251N	Leaky pump [32]
N112D/D116T/Q123D	Na^+^ pump activity abolished [83]	N112D/D116T/Q123E	Similar to N112D/D116T/Q123D [83]
D116E/Q123D	Na^+^ pump activity abolished [10]	P219T/S254A	Red-shifted absorption peak [87] Photocycle slowed [87]

## Data Availability

Data is contained within the article.
